# Reverse transcriptase droplet digital PCR shows high resilience to PCR inhibitors from plant, soil and water samples

**DOI:** 10.1186/s13007-014-0042-6

**Published:** 2014-12-31

**Authors:** Nejc Rački, Tanja Dreo, Ion Gutierrez-Aguirre, Andrej Blejec, Maja Ravnikar

**Affiliations:** Department of Biotechnology and Systems Biology, National Institute of Biology, Večna pot 111, SI-1000 Ljubljana, Slovenia

**Keywords:** PCR amplification, Inhibition, qPCR, Droplet digital PCR, Environmental samples

## Abstract

**Background:**

Detection and quantification of plant pathogens in the presence of inhibitory substances can be a challenge especially with plant and environmental samples. Real-time quantitative PCR has enabled high-throughput detection and quantification of pathogens; however, its quantitative use is linked to standardized reference materials, and its sensitivity to inhibitors can lead to lower quantification accuracy. Droplet digital PCR has been proposed as a method to overcome these drawbacks. Its absolute quantification does not rely on standards and its tolerance to inhibitors has been demonstrated mostly in clinical samples. Such features would be of great use in agricultural and environmental fields, therefore our study compared the performance of droplet digital PCR method when challenged with inhibitors common to plant and environmental samples and compared it with quantitative PCR.

**Results:**

Transfer of an existing Pepper mild mottle virus assay from reverse transcription real-time quantitative PCR to reverse transcription droplet digital PCR was straight forward. When challenged with complex matrices (seeds, plants, soil, wastewater) and selected purified inhibitors droplet digital PCR showed higher resilience to inhibition for the quantification of an RNA virus (Pepper mild mottle virus), compared to reverse transcription real-time quantitative PCR.

**Conclusions:**

This study confirms the improved detection and quantification of the PMMoV RT-ddPCR in the presence of inhibitors that are commonly found in samples of seeds, plant material, soil, and wastewater. Together with absolute quantification, independent of standard reference materials, this makes droplet digital PCR a valuable tool for detection and quantification of pathogens in inhibition prone samples.

**Electronic supplementary material:**

The online version of this article (doi:10.1186/s13007-014-0042-6) contains supplementary material, which is available to authorized users.

## Background

At present, real-time quantitative PCR (qPCR) is the method of choice for detection and quantification of many pathogens and other DNA and RNA targets [1-3]. However, absolute quantification using qPCR depends on the use of standards; i.e., reference materials with known target concentrations, which are rarely commercially available. Even when reference materials are available, quantification accuracy is highly dependent on the amplification efficiencies between the samples and the reference material [4].

One of the factors that can significantly influence amplification efficiency is the presence of inhibitors in samples for PCR amplification. Such inhibitors are either co-extracted with nucleic acids from the samples, or can arise from the extraction procedure itself [5-7]. A wide array of inhibitors (and facilitators) of PCR amplification has been identified over the years, although the mechanisms of inhibition are not always known. Interference with cell lysis, sequestration or degradation of nucleic acids, and hindrance of polymerase activity are all common mechanisms of PCR inhibition [8].

Strategies to lower the influence of inhibitors in qPCR include: (i) extensive sample processing and purification; (ii) decrease in the amount of sample matrix, thereby removing or diluting matrix-derived inhibitors [9]; and (iii) application of corrections during the data analysis. In plant material, and particularly when testing for microorganisms in necrotic plant tissue, sample-specific inhibition is common, and the choice of the optimal DNA extraction and purification procedures can be a lengthy and cumbersome process [10].

Droplet digital PCR (ddPCR) has emerged as a promising technology to overcome these limitations of qPCR. Indeed, ddPCR is an endpoint and absolute measurement approach that enables determination of a target concentration without the need for a standard. This envisages cost-effective precise detection that is accessible to a variety of different scientific fields [11-13]. Moreover, assays developed for qPCR can be readily transferred to the ddPCR platform, with little or no additional optimization [14,15].

Despite relying on the same basic process as PCR and qPCR, i.e., PCR amplification of nucleic acids, it has been proposed that ddPCR can provide higher tolerance to inhibitors present in certain samples [11,13,16,17]. A lower susceptibility of ddPCR to inhibition has been reported for quantification of DNA targets in clinical samples [18], and food and feed samples, using certified reference materials [12]. Dingle et al. [18] reported lower susceptibilities of ddPCR to some inhibitors commonly found in serum, plasma and whole-blood specimens, while Morisset at al. [12] reported that ddPCR is tolerant to inhibitors in maize seed powder.

Compared to the clinical field, the fields of plant analysis and environmental monitoring are faced with a much broader range of target organisms and complex matrices, and the demand for absolute quantification is increasing. Accurate, sensitive and highly specific quantification methods are needed to determine pathogen survival and transmission. In environmental samples in particular, quantification of pathogens is gaining importance, as regulating bodies are shifting toward quantitative microbial risk assessment [6], where the allowed limits are based on the quantities of detected pathogens, and not just on their presence. Absolute quantification in such samples might be hindered by a number of different types and a variable content of inhibitors. Plant metabolites such as neutral and acidic polysaccharides and polyphenolic and humic substances are well-known inhibitors of PCR reactions and are abundant in plant materials, soil and polluted water [5-7,19]. We have previously reported greater tolerance of reverse transcriptase (RT)-ddPCR to inhibitors from wastewater, in comparison to RT-qPCR [15]; however, there are limited data available on the performance of RT-ddPCR in the presence of inhibitors common to diverse plant and environmental samples.

Pepper mild mottle virus (PMMoV) is a plant RNA virus that mainly infects pepper (*Capsicum annum*; Wetter et al., [20]), but also occurs naturally in plants, soil and water. PMMoV can survive the passage through the human digestive tract [21], and has been shown to be commonly present at high concentrations in human feces [22]. PMMoV is also known to persist in the environment for long periods of time [23], and consequently it has been proposed to be a good indicator for fecal contamination of water sources [24]. For these reasons, we chose here a PMMoV assay as a model system to study the effects of inhibitors of PCR in plant and environment matrices.

Using this model system, we compared the resilience of RT-ddPCR and RT-qPCR to selected inhibitors. The RT-ddPCR assay was adapted from an already existing PMMoV qPCR assay [25], and it was evaluated for the detection and quantification of PMMoV in complex matrices found in plants and the environment. In addition, the effects on RT-ddPCR of inhibitors typically present in such matrices were assessed.

## Results

### Transfer of the assay to the ddPCR platform

**Figure 1:**
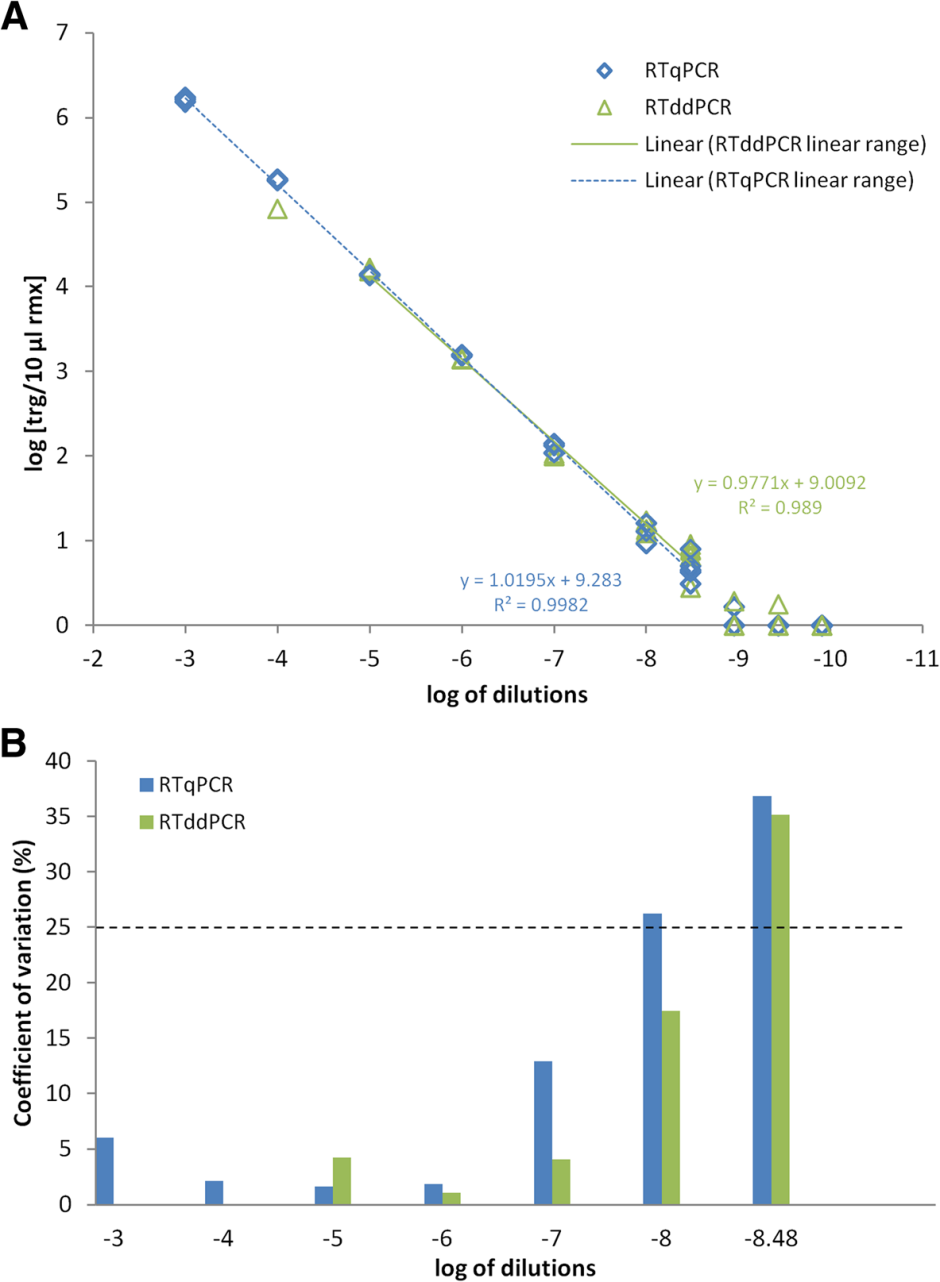
**RT-qPCR and RT-ddPCR parallel analysis of PMMoV RNA serial dilutions. (A)** Correlation between the log serial dilutions and determined concentrations (log target/10 μl reaction mixture) for the RT-qPCR and RT-ddPCR. For each method, the linear regression is plotted, with the equation for the correlation coefficient (R^2^) given. **(B)** Coefficients of variation for the measurement of each of the serial dilutions within the linear range. The dotted horizontal line indicates the maximal acceptable coefficient of variation for the determination of the limit of quantification.

The transfer of the PMMoV assay to RT-ddPCR was successful. RT-qPCR and RT-ddPCR showed similar analytical sensitivities in parallel analysis of PMMoV RNA serial dilutions, (Figure 1 and Additional file 1: Table S1). RT-ddPCR typically showed better precision (smaller coefficients of variation) than RT-qPCR, especially for the dilutions with lower target concentrations (Figure 1B). Both assays showed good linearity of amplification, which was seen as high correlation coefficients (R^2^) obtained by linear regression of the same dilutions (RT-qPCR, 0.998; RT-ddPCR, 0.989) (Figure 1A). Moreover, comparing the slopes of the regression lines showed no significant difference (p > 0.05), an additional argument suggesting that performance of both methods is similar. The dynamic range of quantification for the RT-ddPCR spanned three orders of magnitude (10^4^- 10 target copies/10 μl reaction), which was narrower than that for the RT-qPCR, but with a lower limit of quantification (Figure 1).

### Influence of inhibitors on the different ddPCR parameters

**Figure 2:**
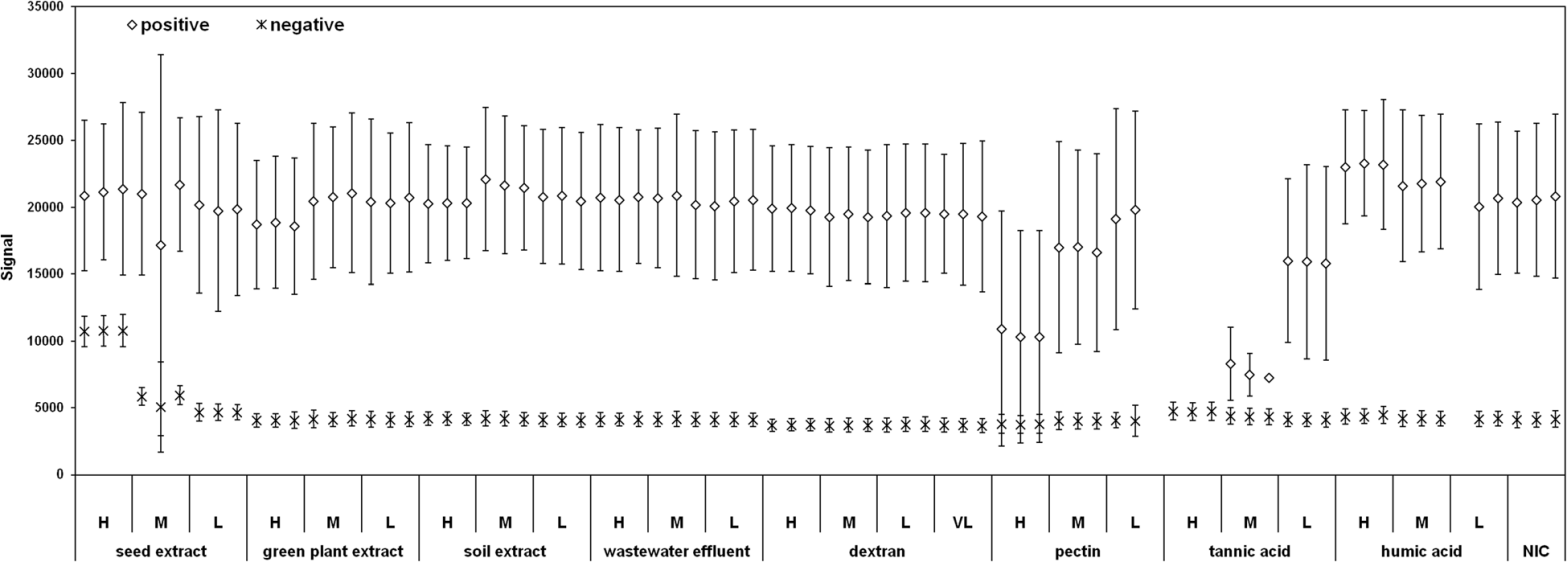
**Mean signals of the positive and negative droplets from the RT-ddPCR for detection of PMMoV in samples spiked with serial dilutions of the inhibitor extracts from the selected matrices and from the chemical inhibitors.** NIC, target RNA with no inhibitors; H, M, L, (VL), high, medium, low (and very low) concentrations of the inhibitor extracts, respectively. Three technical repeats were analyzed for each sample and inhibitor concentration. Error bars represent standard deviation of each signal.

The inhibitors influenced the signals generated in the RT-ddPCR to variable extents. The inhibitors studied here did not considerably interfere with the formation of the droplets. The number of accepted droplets was always above the predefined minimum number (8,000), with the exception of one repetition with added humic acid (7,886 droplets), that was excluded from the subsequent analysis. A mean number of accepted droplets over all of the samples was 12,665 (coefficient of variation, 11%; Additional file 1: Figure S1, top panel). However, some of the inhibitors influenced the RT-ddPCR signals. An increase in the fluorescence of the negative droplets was observed with the seed extract sample, and to a lesser extent in the presence of the higher concentrations of tannic acid (Additional file 1: Figure S2, Figure 2). The observed increases in the fluorescence of negative droplets were most likely due to the inherent fluorescence of seed extract and tannic acid in the channel of the fluorescent dye FAM. The mean signal of positive droplets showed a slightly higher variability among the samples; however, this effect did not always correlate with the concentration of the inhibitors (see, e.g., soil extract in Figure 2; Additional file 1: Figure S3). The signals of the positive and negative droplets were well separated in most cases, as assessed by the mean signals of the negative and positive droplets and their three-times standard deviations (Figure 2). A poorer cluster quality, seen only with the highest concentration of pectin and with the higher concentrations of tannic acid (Additional file 1: Figure S2 B), led to failure in the droplet classification by the Quanta Soft automatic analysis algorithm. The ‘rain’ was low in all cases (maximum 0.01% of accepted droplets; Additional file 1: Figure S1, lower panel). The inhibitors affected the RT-ddPCR in different ways: the seed extract generated an increase in the signal and number of negative droplets and a reduction in the number, but not the signal, of positive droplets; tannic acid generated only a minimal increase in the negative droplet signal, but a marked reduction in the signal and number of positive droplets, which led to their disappearance at the highest inhibitor concentrations; pectin did not have any effects on the signal of negative droplets, nor on the number of positive droplets, but it reduced the signal of the positive droplets, and affected their clustering. These different effects suggest that the different inhibitors can affect the RT-ddPCR process through different mechanisms and at different levels.

**Figure 3:**
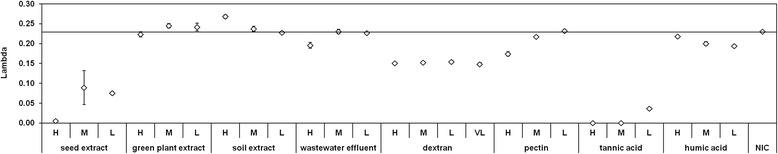
**Mean copies per partition (lambda) determined in the control sample (NIC) with no inhibitor added and in the samples spiked with serial dilutions of the inhibitor extracts from the selected matrices and from the chemical inhibitors.** H, M, L, (VL), high, medium, low (and very low) concentrations of inhibitors, respectively. Black line denotes the mean value of lambda for NIC.

**Figure 4:**
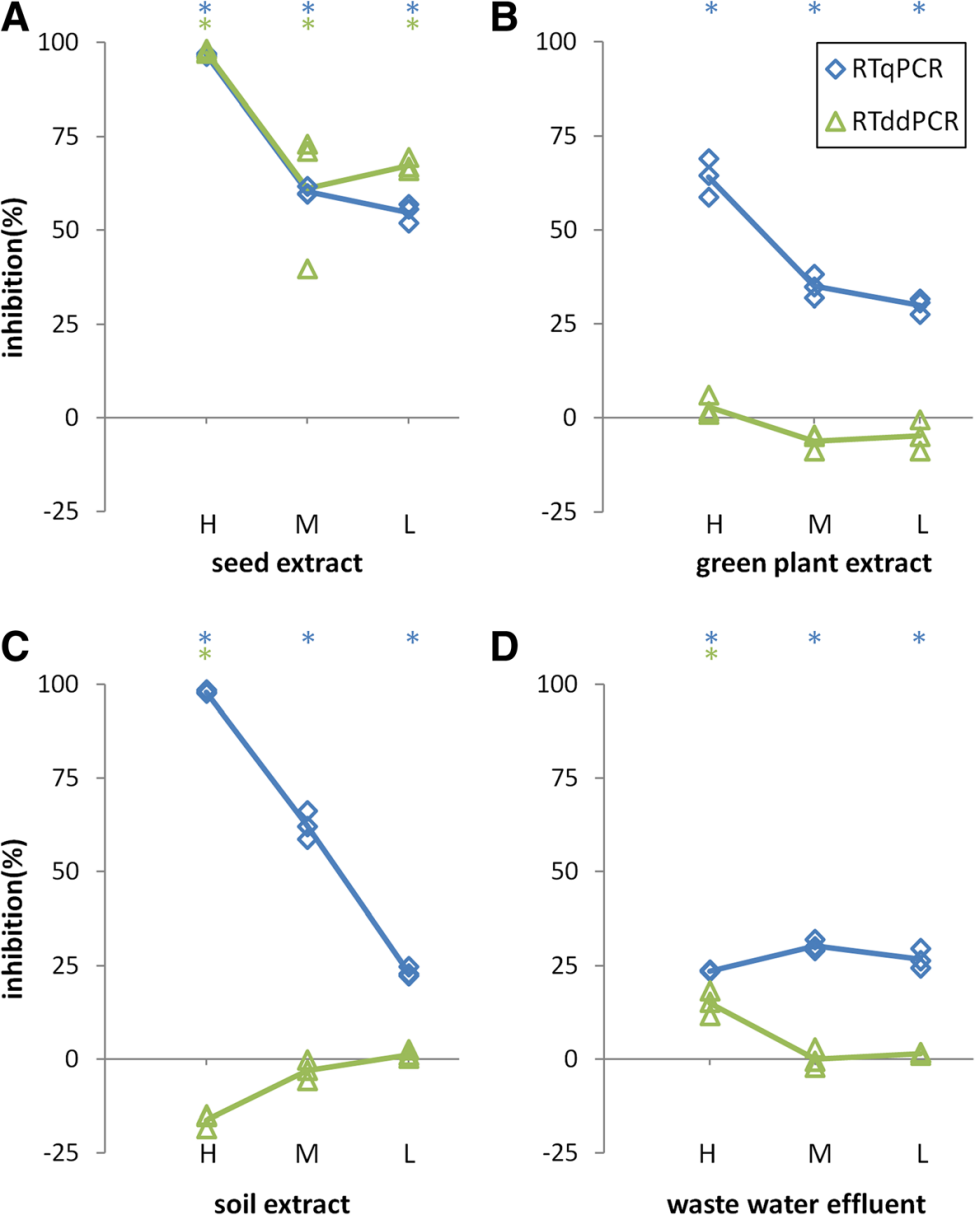
**Influence of the samples spiked with serial dilutions of the inhibitor extracts on the PMMoV quantification by RT-qPCR (blue traces - diamonds) and RT-ddPCR (green traces - triangles).** H, M, L, high, medium, low concentrations of inhibitors, respectively. The PMMoV concentrations are shown relative to those in the no-inhibition control, where 100% inhibition represents total inhibition (no positive signal obtained), and 0% inhibition represents no inhibition (target concentration the same as the no-inhibition control). **(A)** seed extract. **(B)** green plant extract. **(C)** soil extract. **(D)** wastewater effluent. The measurements were carried out in triplicate. Average values of the measurements for each method are connected by traces for better clarity. Asterisks (*) above each chart denote significantly (p < 0.05) different measurements, compared to 0% inhibition (no inhibition control) ± 10%; blue * for RT-qPCR and green * for RT-ddPCR.

**Figure 5:**
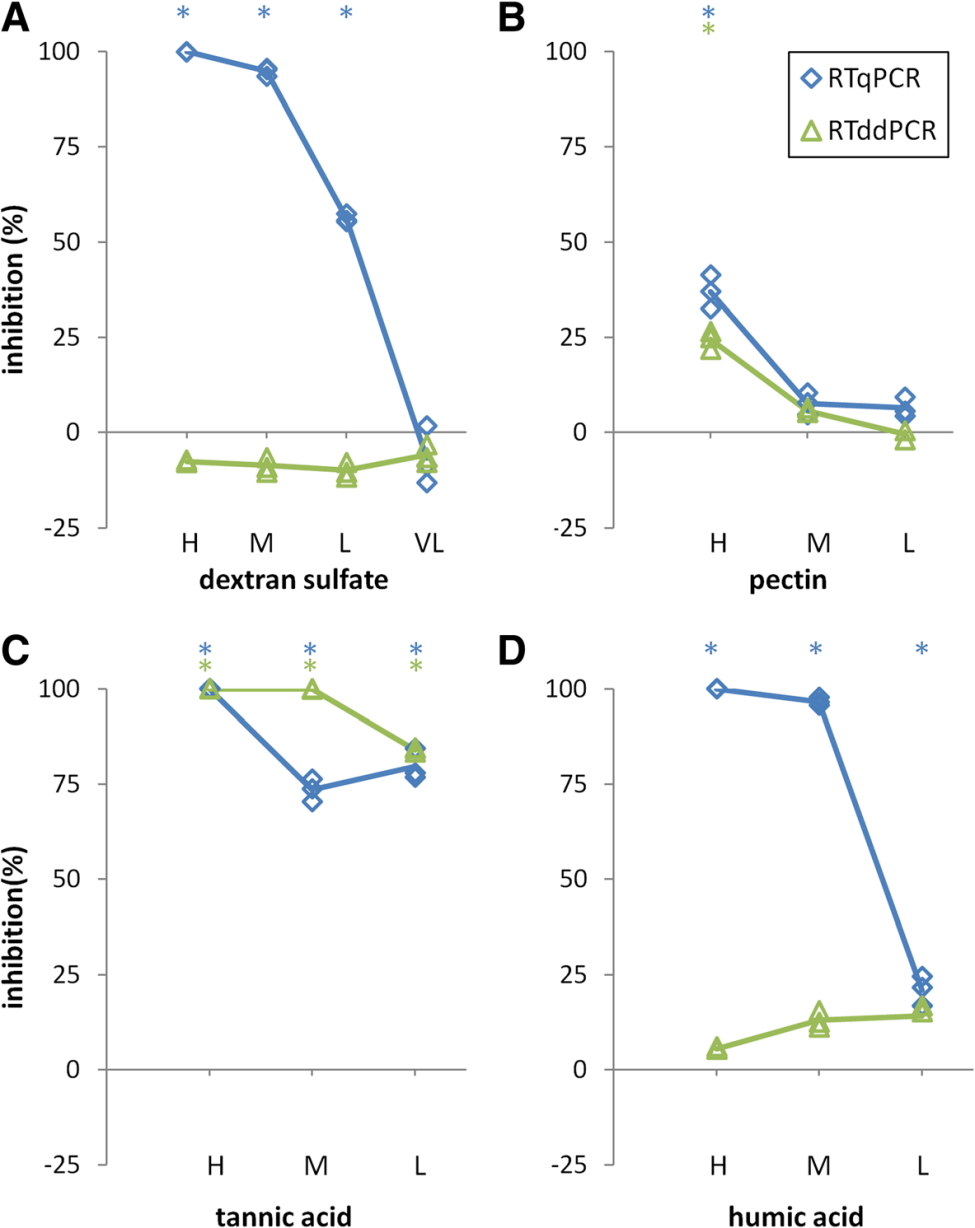
**Influence of the samples spiked with serial dilutions of the chemical inhibitors on the PMMoV quantification by RT-qPCR (blue traces - diamonds) and RT-ddPCR (green traces - triangles).** H, M, L, (VL), high, medium, low (and very low) concentrations of inhibitors, respectively. The PMMoV concentrations are shown relative to those in the no-inhibition control, where 100% inhibition represents total inhibition (no positive signal obtained), and 0% inhibition represents no inhibition (target concentration the same as the no-inhibition control). **(A)** dextran sulfate. **(B)** pectin. **(C)** tannic acid. **(D)** humic acid. The measurements were carried out in triplicate. Average values of the measurements for each method are connected by traces for better clarity. Asterisks (*) above each chart denote significantly (p < 0.05) different measurements, compared to 0% inhibition (no inhibition control) ± 10%; blue *for RT-qPCR and green *for RT-ddPCR.

The mean target copies per partition (droplet) (lambda; [26]) for the RT-ddPCR varied little within replicates for a given inhibitor concentration (average coefficient of variation 7%). However, most of the inhibitors tested led to an underestimation of lambda (Figure 3) in comparison to the no-inhibition control. The seed extract and tannic acid were the inhibitors that most markedly reduced the lambda, compared to the no-inhibition control. This reduction was most likely related to the strong effects exerted by these two inhibitors on the number of positive droplets (Additional file 1: Figure S2), and which resulted in a marked underestimation of the target concentration calculated in these samples (see below, Figures 4A and 5C).

### Influence of inhibitors on the concentrations determined by the RT-qPCR and RT-ddPCR

The target concentrations were calculated for all of the samples with both qPCR and ddPCR. The comparisons between the target concentrations obtained in the absence and presence of different quantities of inhibitors were used to estimate the overall inhibition percentages (Figures 4 and 5), and to compare the inhibitor sensitivities inherent to each of these two methods.

#### Inhibitors from complex matrices

Four matrices were tested for their influence on PCR amplification: seeds, green plant material, soil, and wastewater effluent. The RT-qPCR and RT-ddPCR showed similar susceptibilities to all of the tested concentrations of the seed extract, from total inhibition at the highest tested concentration to partial, but still significant (p < 0.05), inhibition at lower concentrations (Figure 4A). In contrast, green plant extract only affected the RT-qPCR (Figure 4B), as the RT-ddPCR was not significantly inhibited. High concentrations of wastewater effluent partially inhibited both the RT-qPCR and RT-ddPCR. While this inhibition of the RT-qPCR was constant across all of the tested dilutions, the RT-ddPCR was no longer significantly inhibited at the medium and low wastewater effluent concentrations (Figure 4D). The soil extract showed concentration-dependent inhibitory effects on the RT-qPCR. In comparison, the highest concentration of the soil extract significantly (p < 0.05) enhanced the signal of the RT-ddPCR, which led to an overestimation of the target. Medium and low concentrations of this soil extract did not show this effect (Figure 4C).

#### Chemical inhibitors

Pectin and dextran sulfate were selected as representatives of neutral and acidic polysaccharides, respectively [5,19]. Tannic acid was selected as a representative of a polyphenolic substance, and humic acid as a major component of the humic substances found in environmental samples that are rich in plant organic matter [7].

The RT-qPCR and RT-ddPCR showed different sensitivities to these selected chemical inhibitors. At concentrations of dextran sulfate and humic acid where the RT-qPCR was totally inhibited, the RT-ddPCR showed significant tolerance to inhibition (Figure 5). Both the RT-qPCR and RT-ddPCR showed high susceptibilities to inhibition by tannic acid (Figure 5C), whereas pectin only significantly (p < 0.05) affected quantification of the RT-qPCR and RT-ddPCR reactions at the highest tested concentration (Figure 5A).

## Discussion

An RT-qPCR assay that has been used for the detection and quantification of PMMoV, a plant pathogen and a proposed indicator of fecal contamination [27], was successfully transferred to an RT-ddPCR platform. The RT-qPCR and RT-ddPCR both detected low target concentrations; however, the precision of the absolute quantification was greater for the RT-ddPCR. This is in conclusion with our previous experience with assay transfer from RTqPCR to RTddPCR platform [15]. In addition, the absolute quantification in the RT-ddPCR did not rely on reference materials, which is an advantage in many fields for which such materials are difficult to obtain.

Focusing on the RT-ddPCR, these inhibitors affected the monitored parameters differently. In highly inhibited samples, there were no positive drops, which indicated that the amplification was blocked, thus giving a negative result. At lower inhibition levels, the RT-ddPCR signals of positive and/or negative droplets were affected in terms of both the mean values and their distribution (i.e., the standard deviations of the signals). This can lead to failure of the analysis algorithm and lower accuracy of quantification. It has been previously reported that partial inhibition of PCR can lead to increased amounts of rain droplets; i.e., droplets with intermediate fluorescence. In the present study, the assay used for the RT-ddPCR enabled optimal separation of the signals with low levels of rain, whether the inhibitors were present or not. The seed extract and tannic acid affected both the signal levels and the distribution and number of positive droplets, which was reflected in the concentrations determined. Pectin affected the signal and distribution of the positive droplets, but not their number, and therefore the final concentrations were not affected. At higher inhibitor concentrations, humic acid, and more markedly, the soil extract, resulted in higher calculated target concentrations. All of these effects suggest different mechanisms of interference between these inhibitors and the processes in the RT-ddPCR reaction.

Overall, the RT-ddPCR showed higher resilience to inhibitors from the complex samples of the seed extract, plant extract, soil extract, and wastewater effluent than the RT-qPCR, as the inhibition of the RT-ddPCR measurements was typically lower at the same concentrations of inhibitor when compared to RT-qPCR. The RT-ddPCR also showed higher resilience to these selected chemical inhibitors, which represented those that can be commonly found in the complex matrices considered here. This is in agreement with our findings from previous experiments on rotavirus detection in wastewaters [15]. Together with the data of Dingle et al. [18] and Morisset et al. [12], our findings further support the hypothesis that a higher resilience to inhibitors is an inherent characteristic of ddPCR, when compared to qPCR, for both DNA and RNA quantification.

This higher resilience of the RT-ddPCR to such inhibitors can be largely explained by the different methods of signal acquisition between qPCR and ddPCR. qPCR quantification is solely based on the time point within the PCR reaction when the intensity of the fluorescence from the probe degradation increases above background (the cycle of quantification, or Cq; [1]). Due to logarithmic amplification, small differences between reactions are accumulated through each cycle, and these can significantly shift the Cq. Therefore, in qPCR, the efficiency of the primers and the probe annealing, and/or the amplification efficiency, can have large influences on the final results. On the contrary, ddPCR quantifies the target concentration at the end of the amplification, via the Poisson calculation of the ratio between the positive and negative partitions. The time (cycle) when the fluorescent signal in an individual partition increases above background is not relevant for the calculation of the target concentration. Therefore, this partial inhibition can be tolerated [18].

The inhibition observed with the seed extract and soil extract complex samples and with the pectin and tannic acid chemical compounds led to underestimation or overestimation of the target concentrations. These effects were, however, more pronounced for the RT-qPCR. As reported previously by Dingle et al. [18], such inhibitors can influence the mean signals and dispersion of both the positive and negative droplets. However, if the number of positive droplets is not affected and the separation between the positive and negative clusters is still optimal, data analysis can help to mask the inhibition. For example, although the seed extract spiked in the RT-ddPCR reaction increased the background fluorescence, the influence of the middle and lower concentrations of the seed extract on the quantification of the RT-ddPCR can be alleviated by adjustment of the threshold. In a similar way, a decrease in the threshold level allowed correct quantification at higher pectin concentrations.

## Conclusions

Our data confirm a higher resilience of the PMMoV RT-ddPCR to inhibitors that are commonly found in samples of seeds, plant material, soil, and wastewater, in contrast to RT-qPCR. We have also confirmed that different inhibitors affect the ddPCR parameters in a different way, which suggests that there are different mechanisms of interference. Unlike to qPCR, ddPCR data analysis (i.e., the threshold level) can help achieving a correct quantification even in the presence of low to moderate inhibition. In combination with a higher absolute quantification accuracy and the lack of need for standard curve, this makes RT-ddPCR a suitable method for quantification of microbes, being especially beneficial for cases where highly accurate quantification of pathogens in complex matrixes is pursued, i.e., for quantitative microbial risk assessment.

## Materials and methods

### RNA isolation

PMMoV was provided by M. Botermansa and J. (Ko) Th. J. Verhoeven from the National Plant Protection Organization of The Netherlands, and was propagated in pepper (*Capsicum annuum* L*.*) plants under standard greenhouse conditions. RNA from PMMoV-infected pepper plants was isolated using RNeasy Plant Mini kits (Qiagen, CA, USA). Initially, 0.2 g plant material was homogenized in 900 μl RNeasy Plant Mini kit buffer using a FastPrep high-speed benchtop homogenizer (MP Biomedicals, CA, USA) and FastPrep™ Lysing Matrix A (MP Biomedicals) at 4.5 m/s for 40 s, and then incubated for 3 min at 56°C. After centrifugation at 13,000x *g* for 1 min, 550 μl lysate was transferred to a QIAshredder spin column placed in a 2 ml collection tube, and the RNA was isolated following the manufacturer protocol. The purified RNA was eluted in 100 μl molecular grade H_2_O and stored at −20°C until analysis. The RNA was always denatured at 95°C for 5 min and kept on ice until its addition to the reaction.

### Reverse transcription real-time quantitative PCR

The RNA of PMMoV was amplified using the primers and probe described by Haramoto et al. [25]. The reverse transcription and qPCR were combined (i.e., RT-qPCR) into a single step using AgPath-ID™ One-Step RT-PCR kits (Life Technologies, CA, USA). The final reaction volume of 10 μl contained 900 nM primers, 200 nM probe, and 2 μl sample (isolated RNA, or molecular grade RNAse-free water for the no-template controls). Plates were analyzed using a 7900HT Fast Real-Time PCR system (Applied Biosystems, CA, USA). The thermal cycling conditions were as recommended in the AgPath kit manual. The data were acquired and analyzed using the SDS 2.4 software (Applied Biosystems, CA, USA). The threshold was set manually at 0.2 (a level that was above the baseline and sufficiently low to be within the exponential increase region of the amplification curve), and the baseline was set automatically.

### Reverse transcription droplet digital PCR

For the RT-ddPCR reaction, One-Step RT-ddPCR kits for probes (Bio-Rad, CA, USA) were used. Each sample was analyzed in at least triplicate. The final reaction volume of 20 μl contained 900 nM primers, 200 nM probe, and 4 μl sample (isolated RNA, or molecular grade RNAse free water for no-template controls). As the volume of RT-ddPCR reaction was twice the volume of RT-qPCR reaction, to ensure comparable concentrations, all the added reagents and samples were twice the volumes of those added to the RT-qPCR. The further procedures were as described by Rački et al. [15]. The positive droplets that contained amplification products were discriminated from the negative droplets by applying the Quanta Soft automatic analysis of individual wells or by manually selecting a fluorescence threshold (Bio-Rad, CA, USA). The threshold was set to 7000 fluorescence units, except where it was necessary to adjust it to include positive droplets that had shifted due to partial inhibition (Additional file 1: Figure S2). The data generated by the QX 100 droplet reader were rejected from subsequent analysis if a low number of total droplets was accepted (measured) per 20 μL PCR (<8,000), or if all of the droplets were positive (saturation of the reaction).

### Comparison of RT-qPCR and RT-ddPCR performances

A dynamic range of quantification of the PMMoV assays [25] was compared on the qPCR and ddPCR platforms using 10-fold dilutions, and at lower concentrations (3-fold dilutions) of PMMoV RNA in molecular grade water (Sigma, MO, USA). The same RNA dilutions were used for the preparation of reactions for both of these methods. For the comparison of the results, the Cq values for the RT-qPCR and RT-ddPCR concentrations were expressed as copies of target per 10 μl reaction mix (trg/10 μl rmx). For the conversion of Cq to trg/10 μl rmx, the RT-qPCR PMMoV assay had to be calibrated. Due to a lack of availability of a prequantified PMMoV standard, the concentration of PMMoV target copies in the starting preparation of PMMoV RNA (used to prepare the serial dilutions) was calculated from the RT-ddPCR measurements. The most accurately calculated target concentration (lowest coefficient of variation between repetitions, corresponding to the 10^6^ dilution; Figure 1) was chosen and used to calculate back the target concentrations in the rest of the dilutions. Then linear regression (target concentration vs Cq) was used to derive the number of detected targets from the Cq of the samples [28]. The limit of quantification for both of the methods was determined as the lowest dilution of PMMoV RNA that could be quantified with a coefficient of variation between technical repetitions ≤25% [29].

### PCR inhibitors

Two groups of inhibitor sources were selected to evaluate their influence in both RT-qPCR and RT-ddPCR. The first type involved complex matrices that are prone to cause inhibition of PCR; i.e., a seed extract, green plant material, soil and wastewater. The second group involved chemical compounds that are known to be present in plant and/or environmental samples: pectin (from citrus fruit; P9135; Sigma-Aldrich; Germany), dextran sulfate (sodium salt, from *Leuconostoc ssp*.; 31403; Sigma-Aldrich; Germany ), tannic acid (*acidum tanicum*; 16201; Riedel de Haën; Germany) and humic acid (53680; Sigma-Aldrich; Germany). The extracts were prepared as follows: (i) seed extract: 150 *C. annuum* seeds were soaked overnight in 10 ml 0.1 M phosphate buffer at 4°C; (ii) soil extract: 1 g dry planting soil (Archut Fruchstofer Erde; Germany) was soaked overnight in 10 ml 0.1 M phosphate buffer at 4°C; and (iii) green plant extract: 2 g frozen healthy green leaves of *C. annuum* were added to 10 ml 0.1 M phosphate buffer without incubation. Each of the three samples was homogenized with a FastPrep high-speed benchtop homogenizer (MP Biomedicals) at 4.5 m/s for 40 s, using FastPrep™ Lysing Matrix A (MP Biomedicals), and centrifuged at 10,000x *g* for 10 min at 4°C. The supernatants were collected and stored at −20°C until used. Wastewater effluent was obtained from a local wastewater treatment plant (Central Wastewater Treatment Plant Domžale-Kamnik; Ihan, Slovenia) and filtered (Whatman grade 5 H/N paper; Sartorius) and stored as for the extracts. Then, 5-fold serial dilutions of the wastewater effluent and the extracts were prepared to challenge the RT-qPCR and RT-ddPCR reactions. The chemical compound inhibitors were diluted in ultrapure milliQ water (EMD Millipore; MA, USA), as 1% (w/v) solutions of pectin, dextran sulfate, tannic acid and humic acid. Again, 5-fold serial dilutions were added to the RT-qPCR and RT-ddPCR reactions. Where possible, the starting concentration of the inhibitor was selected to be high enough to cause total inhibition of the PMMoV RT-qPCR assay. At least three dilutions were tested for each inhibitor.

### Evaluation of inhibition

The influences of the inhibitors on both the RT-qPCR and RT-ddPCR reactions were assessed relative to the mean measured signals in each sample with no added inhibitors (no-inhibition control, as milliQ water plus PMMoV RNA). The effects of the inhibition on the determined concentrations are given as % inhibition, where 100% inhibition represented total inhibition (no positive signal), and 0% inhibition represented no inhibition (obtained target concentration the same as the no-inhibition control). The same amount of PMMoV RNA was added to each reaction.

Signals obtained from no inhibition control and signals obtained from reactions, challenged with different inhibitor concentrations (Figures 4 and 5) were tested using the general linear hypothesis method (R package multcomp, [30]).

However, because of high repeatability of the results, all the differences were statistically significant (p < 0.05), even the ones that in practical term were the same. Therefore to give statistical analysis some practical relevance, tolerance of 0% inhibition ±10% was introduced i.e. a difference of measured signal was considered relevant, if it was significantly (p < 0.05) different from the signal obtained from no inhibition control ±10%.

To define the influence of the inhibitors on the different parameters for the RT-ddPCR, the key parameters likely to be affected by the presence of the inhibitors were monitored: (i) signal levels and distribution; and (ii) determined concentrations. The percentage of the ‘rain’ droplets out of all of the accepted droplets was derived from the number of droplets with signals in the range between the mean amplitude of the negative droplets plus three standard deviations, and the mean amplitude of the positive droplets minus three standard deviations; i.e., the droplets outside the majority of the normally distributed signals.

## Additional files

Additional file 1: Table S1. Performance of PMMoV assay by RT-qPCR and RT-ddPCR. Measurements of dilutions of target PMMoV RNA by both platforms are presented. **Figure S1.** Number of accepted droplets in each reaction (top), percent of droplets with intermediate fluorescence (‘rain’; bottom), as influenced by the spiked serial dilutions of the inhibitor extracts from the selected matrices and from the chemical inhibitors in the RT-ddPCR quantification of PMMoV. Control, target RNA with no inhibitors; bias, systematic distortion; H, M, L (L*), high, medium, low (and lowest) concentrations of inhibitor, respectively. Black line denotes the minimal number of accepted droplets (8000) that had to be obtained in order for measurement to be considered valid. **Figure S2.** Signals for the ranges of the positive droplets, as influenced by the spiked serial dilutions of the inhibitor extracts from the selected matrices and from the chemical inhibitors in the RT-ddPCR quantification of PMMoV. Darker colors correspond to higher inhibitor concentrations. On the bottom no-template control (NTC) and no inhibition control (NIC) are shown. **Figure S3.** Droplet plots of the representative RT-ddPCR reactions spiked with serial dilutions of the inhibitor extracts (see legend) from the selected matrices (A) and from the chemical inhibitors (B). For each dilution of the inhibitors, only one of three repetitions is shown. Grey droplets, negative for PMMoV; blue droplets, positive for PMMoV; pink line, position of the threshold for each sample. For easier comparison of the influence of each inhibitor on the amplitude plots, the corresponding no inhibition control (NIC) and no-template control (NTC) are shown for every plot.
