# Supplementary material for: Reverse transcriptase droplet digital PCR shows high resilience to PCR inhibitors from plant, soil and water samples
Source: Plant Methods. 2014 Dec 31;10:42. doi: 10.1186/s13007-014-0042-6 (PMC4307183; doi:10.1186/s13007-014-0042-6)
Supplement: Additional file 1: Table S1. — Performance of PMMoV assay by RT-qPCR and RT-ddPCR. Measurements of dilutions of target PMMoV RNA by both platforms are presented. Figure S1. Number of accepted droplets in each reaction (top), percent of droplets with intermediate fluorescence (‘rain’; bottom), as influenced by the spiked serial dilutions of the inhibitor extracts from the selected matrices and from the chemical inhibitors in the RT-ddPCR quantification of PMMoV. Control, target RNA with no inhibitors; bias, systematic distortion; H, M, L (L*), high, medium, low (and lowest) concentrations of inhibitor, respectively. Black line denotes the minimal number of accepted droplets (8000) that had to be obtained in order for measurement to be considered valid. Figure S2. Signals for the ranges of the positive droplets, as influenced by the spiked serial dilutions of the inhibitor extracts from the selected matrices and from the chemical inhibitors in the RT-ddPCR quantification of PMMoV. Darker colors correspond to higher inhibitor concentrations. On the bottom no-template control (NTC) and no inhibition control (NIC) are shown. Figure S3. Droplet plots of the representative RT-ddPCR reactions spiked with serial dilutions of the inhibitor extracts (see legend) from the selected matrices (A) and from the chemical inhibitors (B). For each dilution of the inhibitors, only one of three repetitions is shown. Grey droplets, negative for PMMoV; blue droplets, positive for PMMoV; pink line, position of the threshold for each sample. For easier comparison of the influence of each inhibitor on the amplitude plots, the corresponding no inhibition control (NIC) and no-template control (NTC) are shown for every plot. [file 13007_2014_42_MOESM1_ESM.docx]

Plant methods

Supplementary Material

**Reverse transcriptase droplet digital PCR shows high resilience to PCR inhibitors from plant, soil and water samples**

Nejc Rački, Tanja Dreo, Ion Gutierrez Aguirre, Andrej Blejec and Maja Ravnikar

**Corresponding author: Nejc Rački**

Department of Biotechnology and Systems Biology,

National Institute of Biology

Večna pot 111

SI-1000 Ljubljana, Slovenia

Email: nejc.racki@nib.si

Tel: +386-5-9232833

**Table S1. Performance of PMMoV assay by RT-qPCR and RT-ddPCR.** Measurements of dilutions of target PMMoV RNA by both platforms are presented.

| **Dilution** | **RT-qPCR** | | | | |  | **RT-ddPCR** | | | | | |
| --- | --- | --- | --- | --- | --- | --- | --- | --- | --- | --- | --- | --- |
| **(fold)** | **Cq** | **Conc.**  **(trg/ 10 μl rmx)** | **Mean** | **Standard deviation** | **Coefficient of variation (%)** |  | **Accepted**  **droplets** | **Positives** | **Conc.**  **(trg/ 10 μl rmx)** | **Mean** | **Standard deviation** | **Coefficient of variation (%)** |
| **1.E+03** | 15.07 | 1644941.2 | 1625382.3 | 98125.0 | 6.0 |  | Over saturation point, therefore not analyzed | | | | | |
|  | 15.00 | 1712254.9 |  |  |  |  |  |  |  |  |  |  |
|  | 15.19 | 1518950.9 |  |  |  |  |  |  |  |  |  |  |
| **1.E+04** | 18.54 | 187852.2 | 183351.6 | 3975.0 | 2.2 |  | 13545 | 13545 | 10000000 | Already saturated | | |
|  | 18.59 | 181881.4 |  |  |  |  | 10610 | 10610 | 10000000 |  |  |  |
|  | 18.60 | 180321.1 |  |  |  |  | 13526 | 13519 | 83100 | n.a. | n.a. | n.a. |
| **1.E+05** | 22.70 | 13932.2 | 13723.3 | 221.1 | 1.6 |  | 12040 | 9364 | 16500 | 15733.3 | 665.8 | 4.2 |
|  | 22.75 | 13491.8 |  |  |  |  | 12106 | 9125 | 15400 |  |  |  |
|  | 22.72 | 13745.9 |  |  |  |  | 10768 | 8083 | 15300 |  |  |  |
| **1.E+06** | 26.19 | 1573.9 | 1560.8 | 28.9 | 1.9 |  | 12085 | 1464 | 1420 | 1406.7 | 15.3 | 1.1 |
|  | 26.18 | 1580.9 |  |  |  |  | 11330 | 1342 | 1390 |  |  |  |
|  | 26.23 | 1527.7 |  |  |  |  | 10952 | 1315 | 1410 |  |  |  |
| **1.E+07** | 30.07 | 138.6 | 126.7 | 16.3 | 12.9 |  | 11561 | 112 | 107 | 102.3 | 4.2 | 4.1 |
|  | 30.47 | 108.1 |  |  |  |  | 11353 | 104 | 101 |  |  |  |
|  | 30.13 | 133.5 |  |  |  |  | 11039 | 99 | 99 |  |  |  |
| **1.E+08** | 34.39 | 9.3 | 12.7 | 3.3 | 26.2 |  | 12184 | 19 | 17.1 | 14.3 | 2.5 | 17.5 |
|  | 33.89 | 12.7 |  |  |  |  | 12239 | 15 | 13.5 |  |  |  |
|  | 33.53 | 16.0 |  |  |  |  | 10755 | 12 | 12.3 |  |  |  |
| **3.E+08** | 35.37 | 5.0 | 5.0 | 1.8 | 36.8 |  | 13198 | 8 | 6.7 | 6.8 | 2.4 | 35.2 |
|  | 35.56 | 4.5 |  |  |  |  | 9941 | 7 | 7.7 |  |  |  |
|  | 34.64 | 8.0 |  |  |  |  | 11001 | 9 | 9.0 |  |  |  |
|  | 36.17 | 3.1 |  |  |  |  | 11761 | 3 | 2.8 |  |  |  |
|  | 35.65 | 4.2 |  |  |  |  | 12402 | 9 | 8.0 |  |  |  |
| **9.E+08** | n.d. | n.d. | n.a. | n.a. | n.a. |  | 12191 | 0 | n.d. | n.a. | n.a. | n.a. |
|  | 38.15 | 0.9 |  |  |  |  | 9641 | 0 | n.d. |  |  |  |
|  | 38.03 | 1.0 |  |  |  |  | 11383 | 0 | n.d. |  |  |  |
|  | 37.17 | 1.6 |  |  |  |  | 11999 | 0 | n.d. |  |  |  |
|  | n.d. | n.d. |  |  |  |  | 11462 | 2 | 1.9 |  |  |  |
| **2.7E+09** | n.d. | n.d. | n.a. | n.a. | n.a. |  | 12360 | 2 | 1.8 | n.a. | n.a. | n.a. |
|  | n.d. | n.d. |  |  |  |  | 11380 | 1 | 1.0 |  |  |  |
|  | n.d. | n.d. |  |  |  |  | 11600 | 0 | n.d. |  |  |  |
|  | 38.05 | 0.9 |  |  |  |  | 11308 | 0 | n.d. |  |  |  |
|  | n.d. | n.d. |  |  |  |  | 12039 | 0 | n.d. |  |  |  |
| **8.1E+09** | n.d. | n.d. | n.a. | n.a. | n.a. |  | 12067 | 0 | n.d. | n.a. | n.a. | n.a. |
|  | n.d. | n.d. |  |  |  |  | 10389 | 0 | n.d. |  |  |  |
|  | n.d. | n.d. |  |  |  |  | 10977 | 0 | n.d. |  |  |  |
|  | n.d. | n.d. |  |  |  |  | 10604 | 0 | n.d. |  |  |  |
|  | n.d. | n.d. |  |  |  |  | 10943 | 0 | n.d. |  |  |  |
| No-template control | n.d. | n.d. | n.a. | n.a. | n.a. |  | 11501 | 0 | n.d. | n.a. | n.a. | n.a. |
|  | n.d. | n.d. |  |  |  |  | 9884 | 0 | n.d. |  |  |  |
|  | n.d. | n.d. |  |  |  |  | 11043 | 0 | n.d. |  |  |  |
|  | n.d. | n.d. |  |  |  |  | 11793 | 0 | n.d. |  |  |  |
|  | n.d. | n.d. |  |  |  |  | 11905 | 0 | n.d. |  |  |  |

Cq, cycle of quantification; Conc., concentration; trg/10 μl rmx, copies of target per 10 µl reaction mixture; n.d., not detected; n.a., not applicable; saturation point, concentration of target where all ddPCR droplets are positive; rnx - reaction

**Figure S1. Number of accepted droplets in each reaction (top), percent of droplets with intermediate fluorescence (‘rain’; bottom), as influenced by the spiked serial dilutions of the inhibitor extracts from the selected matrices and from the chemical inhibitors in the RT-ddPCR quantification of PMMoV.** Control, target RNA with no inhibitors; bias, systematic distortion; H, M, L (L*), high, medium, low (and lowest) concentrations of inhibitor, respectively. Black line denotes the minimal number of accepted droplets (8000) that had to be obtained in order for measurement to be considered valid.

**
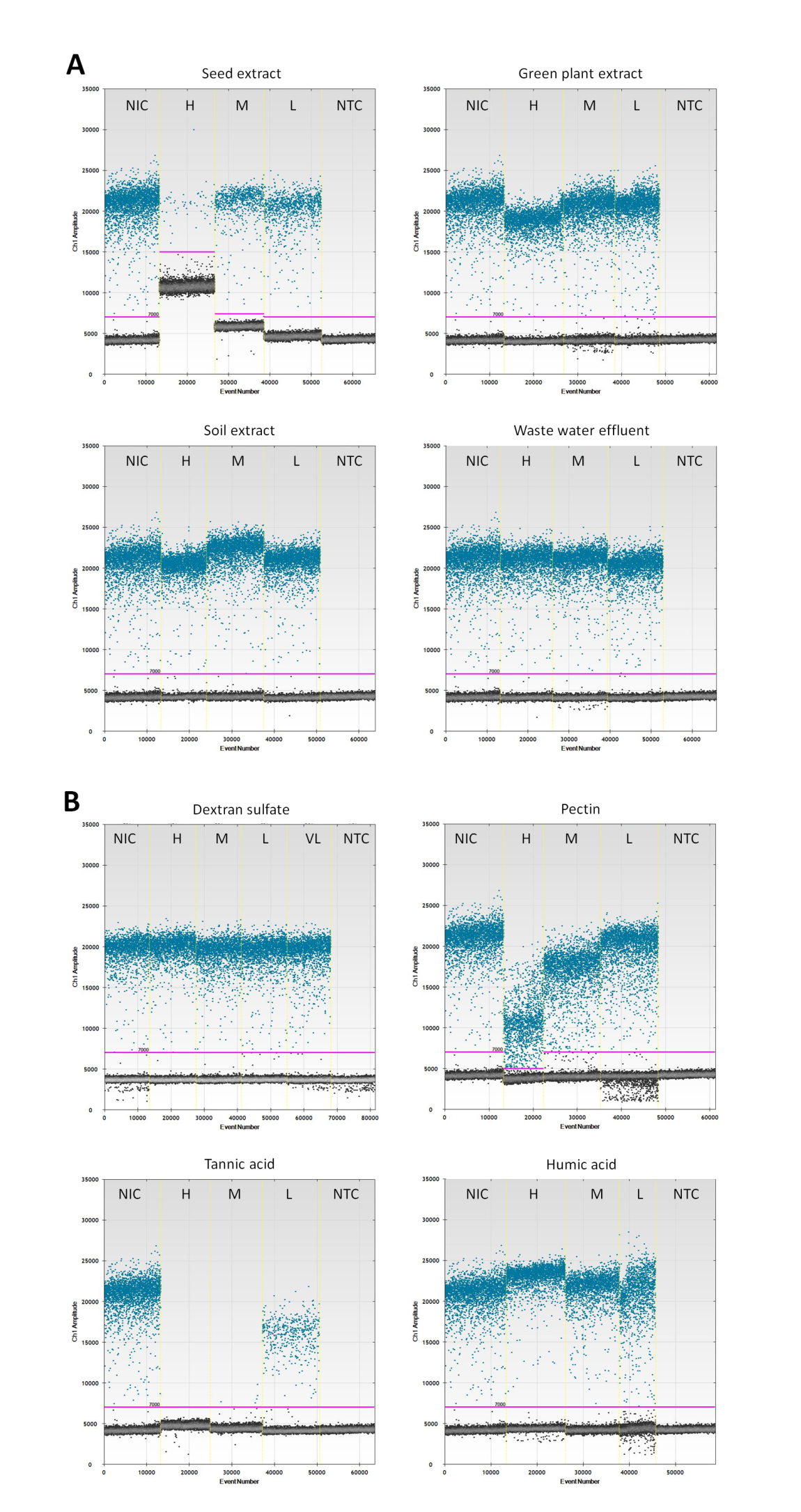
**

**Figure S2. Droplet plots of the representative RT-ddPCR reactions spiked with serial dilutions of the inhibitor extracts (see legend) from the selected matrices (A) and from the chemical inhibitors (B).** For each dilution of the inhibitors, only one of three repetitions is shown. Grey droplets, negative for PMMoV; blue droplets, positive for PMMoV; pink line, position of the threshold for each sample. For easier comparison of the influence of each inhibitor on the amplitude plots, the corresponding no inhibition control (NIC) and no-template control (NTC) are shown for every plot.

| **Fig. S2 legend:** | |  |
| --- | --- | --- |
|  |  |  |
| Panel  **A** | Inhibitor | Concentration  (v/v) |
| H | Seed extract | 1% |
| M |  | 0.2% |
| L |  | 0.04% |
| H | Green plant  extract | 1% |
| M |  | 0.2% |
| L |  | 0.04% |
| H | Soil extract | 1% |
| M |  | 0.2% |
| L |  | 0.04% |
| H | Wastewater  effluent | 2% |
| M |  | 0.4% |
| L |  | 0.08% |
|  |  |  |
| Panel  **B** | Inhibitor | Concentration (w/v) |
| H | Dextran sulfate | 0.0002% |
| M |  | 0.00004% |
| L |  | 0.000008% |
| VL |  | 0.0000016% |
| H | Pectin | 0.1% |
| M |  | 0.02% |
| L |  | 0.004% |
| H | Tannic acid | 0.01% |
| M |  | 0.002% |
| L |  | 0.0004% |
| H | Humic acid | 0.002% |
| M |  | 0.0004% |
| L |  | 0.00008% |
|  |  |  |
|  |  |  |
| Controls | | |
| NIC | No inhibition control | |
| NTC | No template control | |


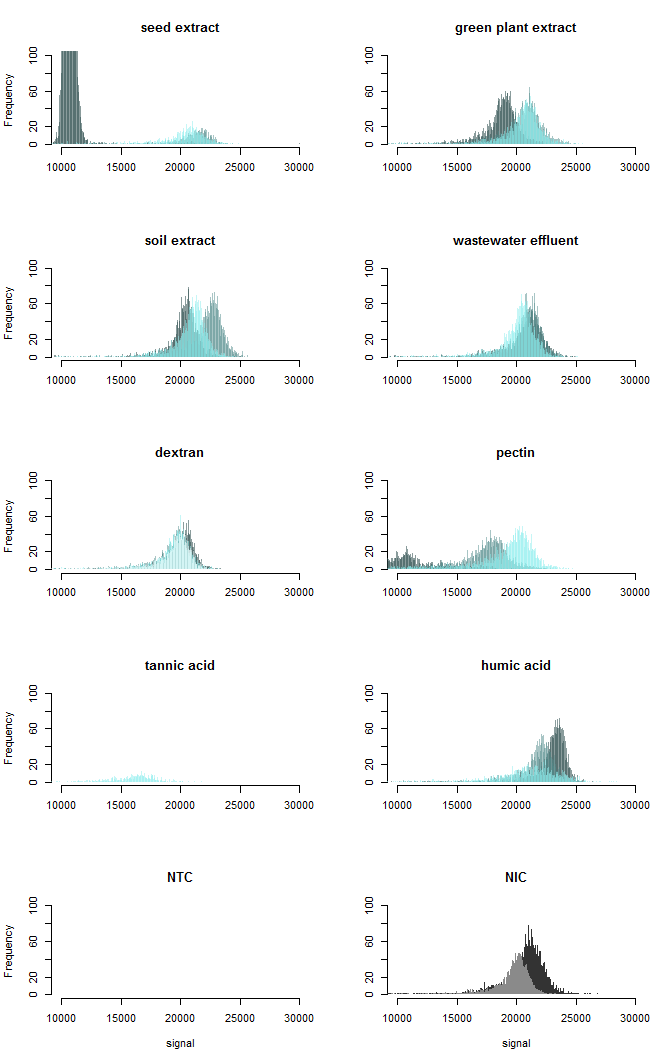


**Figure S3. Signals for the ranges of the positive droplets, as influenced by the spiked serial dilutions of the inhibitor extracts from the selected matrices and from the chemical inhibitors in the RT-ddPCR quantification of PMMoV.** Darker colors correspond to higher inhibitor concentrations. On the bottom no-template control (NTC) and no inhibition control (NIC) are shown.
